# Ruptured Appendiceal Diverticulum Presenting as Appendiceal Phlegmon Mimicking Acute Appendicitis: A Case Report

**DOI:** 10.7759/cureus.105399

**Published:** 2026-03-17

**Authors:** Mohammad H Ababneh, Haneen A Noures, Yacoub H Alawneh, Deema I Isbeitan, Farah K Dabaibeh

**Affiliations:** 1 Department of Surgery, Royal Medical Services, Amman, JOR; 2 Department of Pathology, Royal Medical Services, Amman, JOR

**Keywords:** appendiceal neoplasms, appendicitis, case reports, diverticulitis, diverticulosis, diverticulosis of the appendix, diverticulum, pseudodiverticula, ruptured appendiceal diverticulum

## Abstract

Appendiceal diverticulosis is a rare condition, with a reported incidence ranging from approximately 0.004% to 2.1%, and it may remain clinically silent or present with inflammation that closely resembles acute appendicitis. Distinguishing appendiceal diverticulitis from typical appendicitis preoperatively is often challenging because clinical manifestations and imaging findings are frequently nonspecific and may suggest an appendiceal phlegmon or localized perforation rather than a separate diverticular pathology. Definitive diagnosis is usually established by histopathological examination following appendectomy, which is also essential for assessing perforation and for excluding associated neoplastic changes.

We report the case of a 34-year-old female who presented with right lower quadrant abdominal pain, nausea, and clinical findings consistent with acute appendicitis. Radiological evaluation suggested appendiceal inflammation with associated phlegmon formation. The patient underwent surgical intervention, and intraoperative findings revealed a ruptured appendiceal diverticulum with adjacent inflammatory changes. Histopathological examination confirmed perforated appendiceal diverticulitis with no evidence of malignancy. The postoperative course was uneventful, and the patient recovered well. This report highlights the importance of considering appendiceal diverticulitis in the differential diagnosis of acute right lower quadrant pain and emphasizes the role of careful histopathological evaluation after appendectomy to ensure accurate diagnosis and appropriate management.

## Introduction

Appendiceal diverticulosis is a rare disease, usually asymptomatic, characterized by an outpouching of the appendiceal wall that may be either acquired or congenital [1,2]. The majority of appendiceal diverticula are acquired (pseudodiverticula) and result from either inflammatory or noninflammatory processes [3]. The former arises from weakening of the appendiceal wall due to inflammation, whereas the latter occurs because of increased intraluminal pressure caused by obstruction (stricture, fecalith, tumors, etc.) and muscular contraction [3]. Congenital appendiceal diverticulosis is believed to be associated with chromosomal anomalies, such as trisomy 13 or trisomy 21; additional causes include developmental anomalies. This condition is clinically significant because it mimics appendicitis in symptoms and has a much higher rate of perforation (30-70%) compared to typical appendicitis [3,4]. It is also associated with a higher risk of appendiceal neoplasms [3,4].

## Case presentation

A 35-year-old married woman with no known chronic medical illnesses and no previous surgical history presented to the emergency department with a three-day history of worsening pain in the right lower quadrant of the abdomen. The pain began gradually and was described as intermittent, varying from sharp to dull, and remained localized to the right lower abdomen. It was associated with fever, anorexia, and nausea. She denied vomiting, changes in bowel habits, urinary symptoms, or gynecological complaints. On admission, her vital signs revealed a temperature of 38 °C, blood pressure of 120/60 mmHg, heart rate of 94 beats per minute, and oxygen saturation within normal limits on room air. Abdominal examination revealed tenderness localized to the right iliac fossa with positive rebound tenderness. Psoas and obturator signs were positive. No palpable mass was found.

Initial laboratory investigations revealed leukocytosis with a white blood cell (WBC) count of 12 × 10³/µL and neutrophilia (71.7%). Other laboratory parameters were within normal limits. The clinical picture was highly suggestive of acute appendicitis, with a high likelihood according to the Alvarado scoring system. Although an Appendicitis Inflammatory Response (AIR) score was considered, C-reactive protein (CRP) levels were not available at the time of assessment.

**Figure 1:**
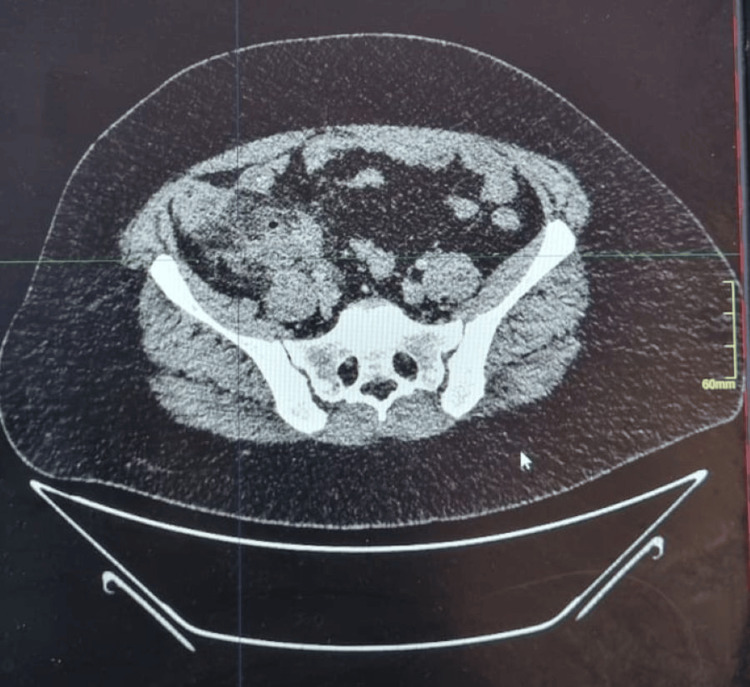
Preoperative contrast-enhanced CT of the abdomen and pelvis demonstrating significant inflammatory changes in the right iliac fossa CT: computed tomography

**Figure 2:**
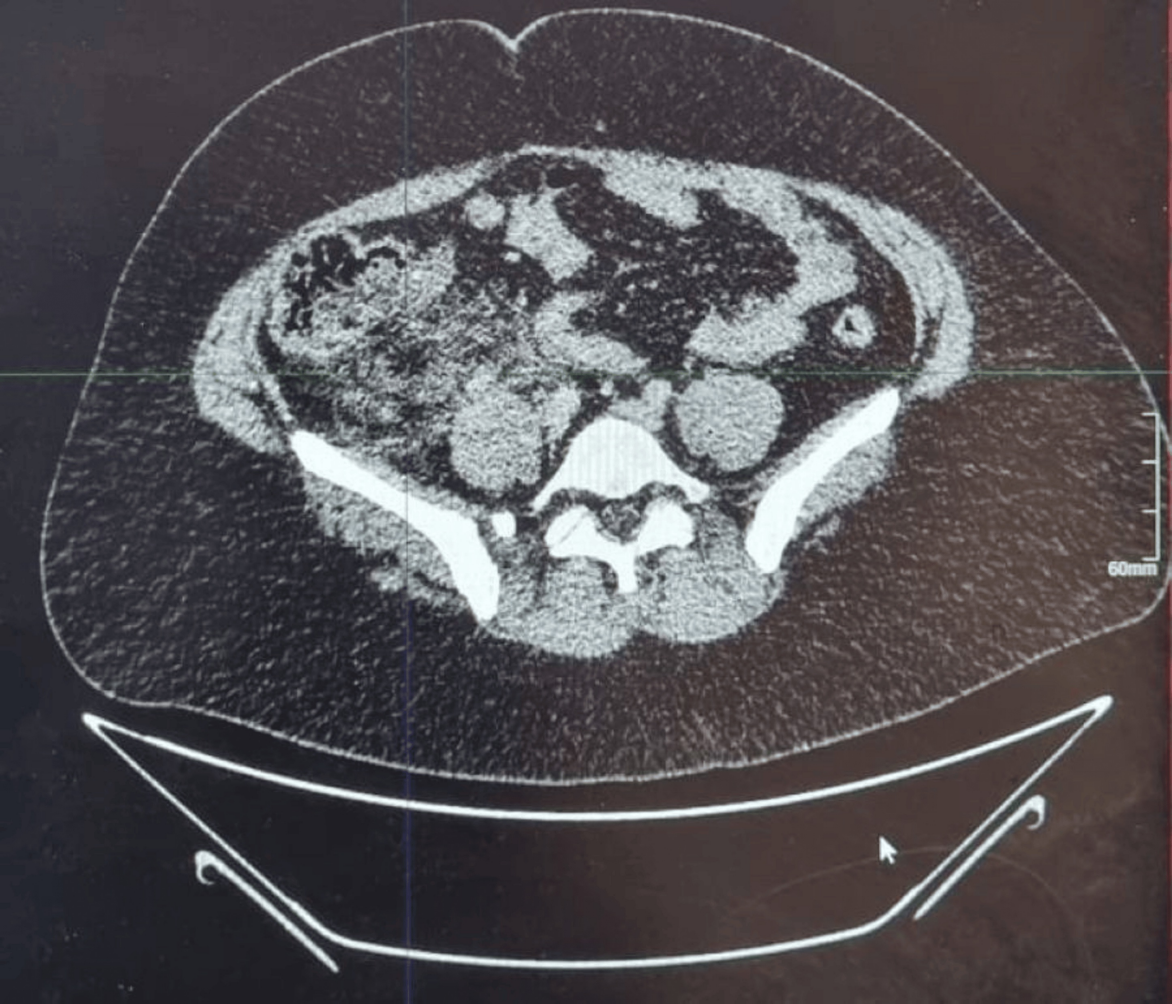
Another preoperative contrast-enhanced CT of the abdomen and pelvis revealed thickening of the adjacent cecal wall and terminal ileum, suggesting a localized inflammatory process A periappendiceal gas locule and a small amount of adjacent free fluid were also observed CT: computed tomography

Preoperative contrast-enhanced CT of the abdomen and pelvis showed significant inflammatory changes in the right iliac fossa (Figure 1). Axial images revealed a poorly defined, heterogeneous soft-tissue mass in the periappendiceal region with extensive fat stranding. Thickening of the adjacent cecal wall and terminal ileum was noted, suggesting a localized inflammatory process. A periappendiceal gas locule and mild adjacent free fluid were also identified (Figure 2). These findings were consistent with complicated appendicitis with phlegmon formation. Based on the clinical presentation and radiological findings, the decision was made to perform surgical intervention.

**Figure 3:**
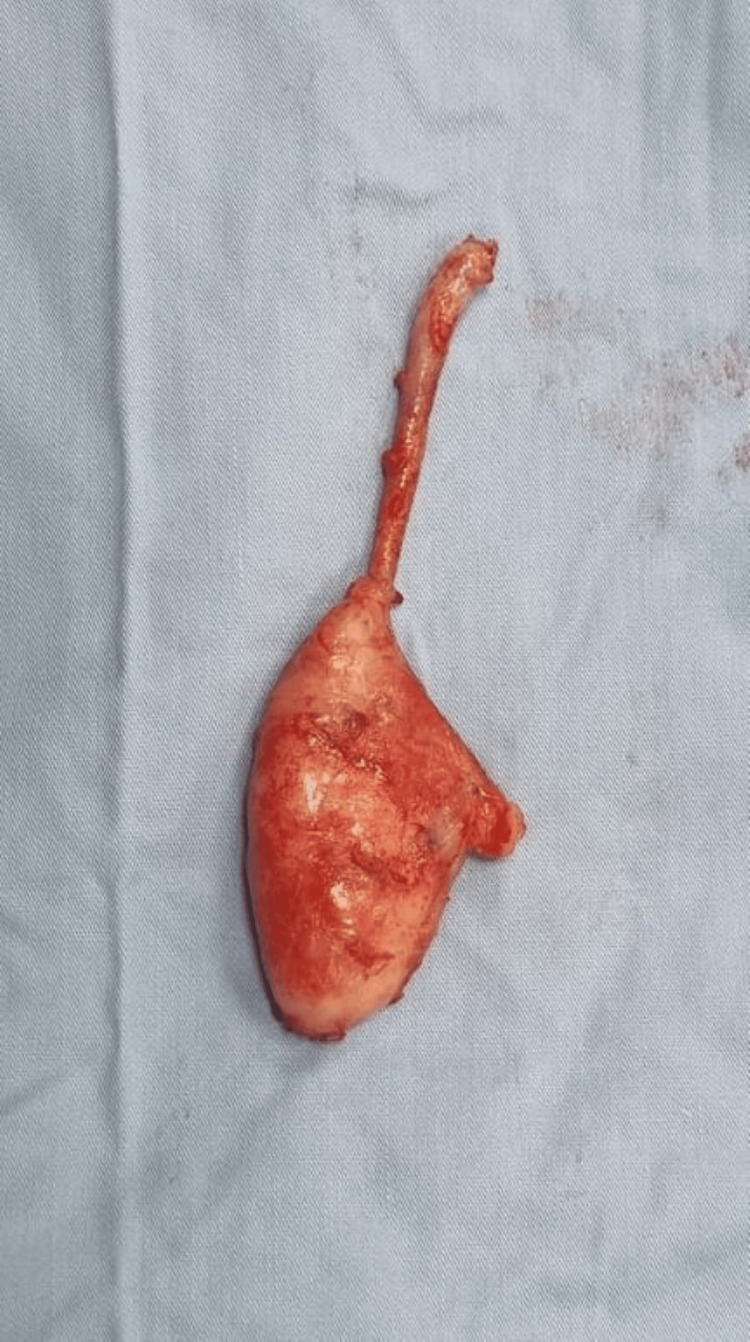
Postoperative gross appearance: 10 cm appendix with phlegmon at the tip

An open appendectomy was performed. Intraoperatively, the appendix was inflamed and adherent to the terminal ileum and pelvic structures, with phlegmon formation at the distal tip. No obvious perforation or purulent discharge was observed. Minimal adjacent free fluid was present. The appendiceal base appeared healthy. The procedure was completed smoothly with adequate hemostasis and no intraoperative complications. The gross appearance of the specimen is shown in Figure 3.

Postoperatively, the patient showed consistent clinical improvement. She was afebrile, tolerated oral intake, ambulated well, and reported only mild incisional pain. On postoperative day two, a laboratory report revealed a significant drop in packed cell volume (PCV) to 20% from a preoperative value of 31.5%. Despite this result, the patient remained hemodynamically stable with no clinical signs of bleeding. An urgent abdominal CT angiography was performed, and demonstrated no evidence of active bleeding or contrast extravasation. Repeat complete blood count testing showed a PCV of 29%, and a subsequent repeat before discharge was 32.2%, confirming that the initial low value was most likely a pre-analytical error, such as hemodilution from IV fluids or a sampling error, and no further intervention was required.

The WBC count temporarily rose postoperatively to 14 × 10³/µL before decreasing to 9 × 10³/µL before discharge, with neutrophil percentage returning to normal at 59%. The patient was discharged home in stable condition with instructions and a follow-up appointment scheduled for two weeks later. At follow-up, she remained asymptomatic. Physical examination showed a clean surgical wound and a soft, non-tender abdomen.

**Figure 4:**
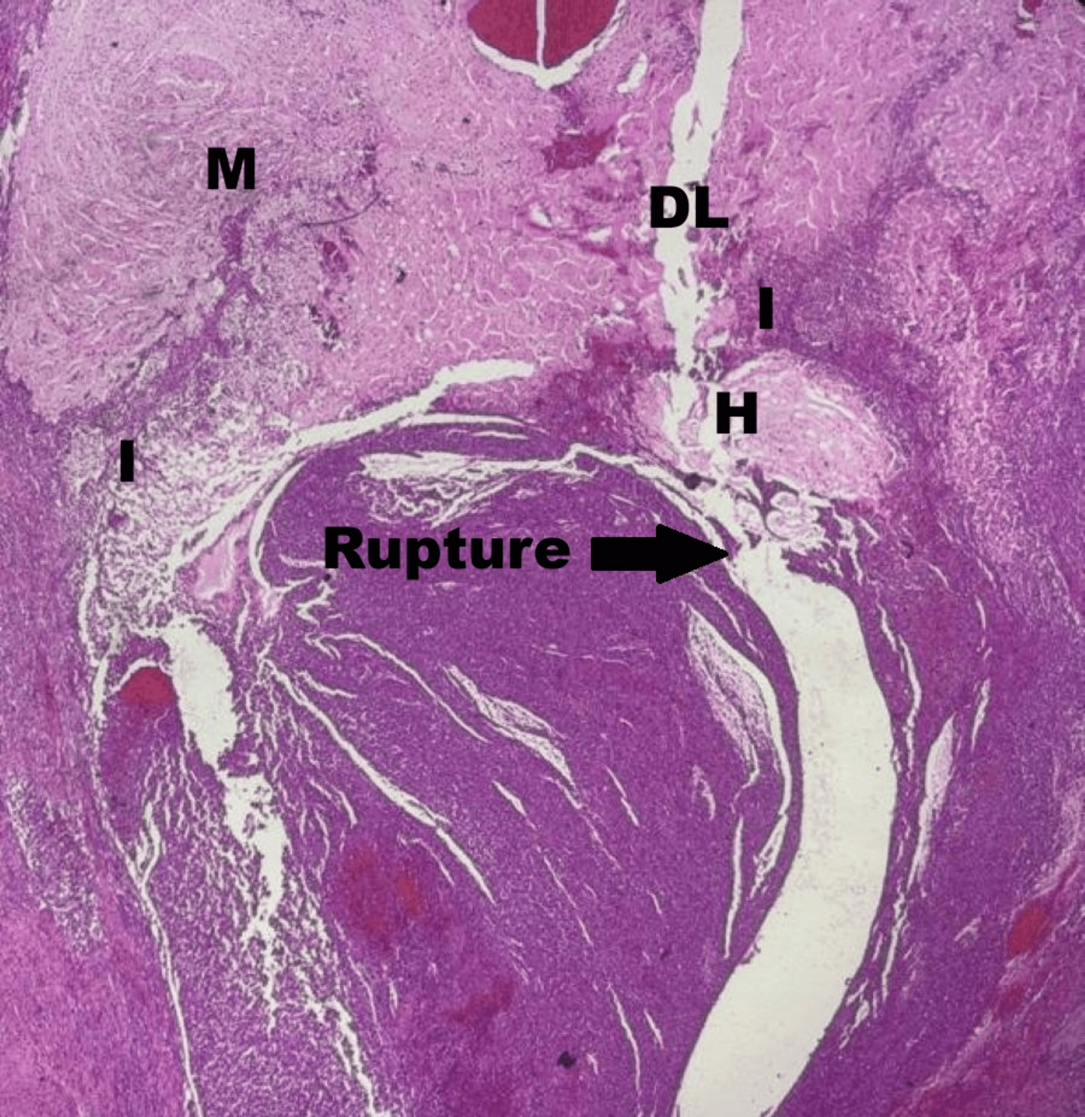
Histopathological section of the appendix (H&E stain, low power) - image 1 The image confirms a ruptured appendiceal diverticulum. Key landmarks include the herniated mucosa (H) protruding through a defect in the muscularis propria into the diverticular lumen (DL). Extensive mucin extravasation (M) is seen in the periappendiceal space, accompanied by a dense inflammatory infiltrate (I) and reactive fibrosis. No evidence of cellular atypia or malignancy is identified

**Figure 5:**
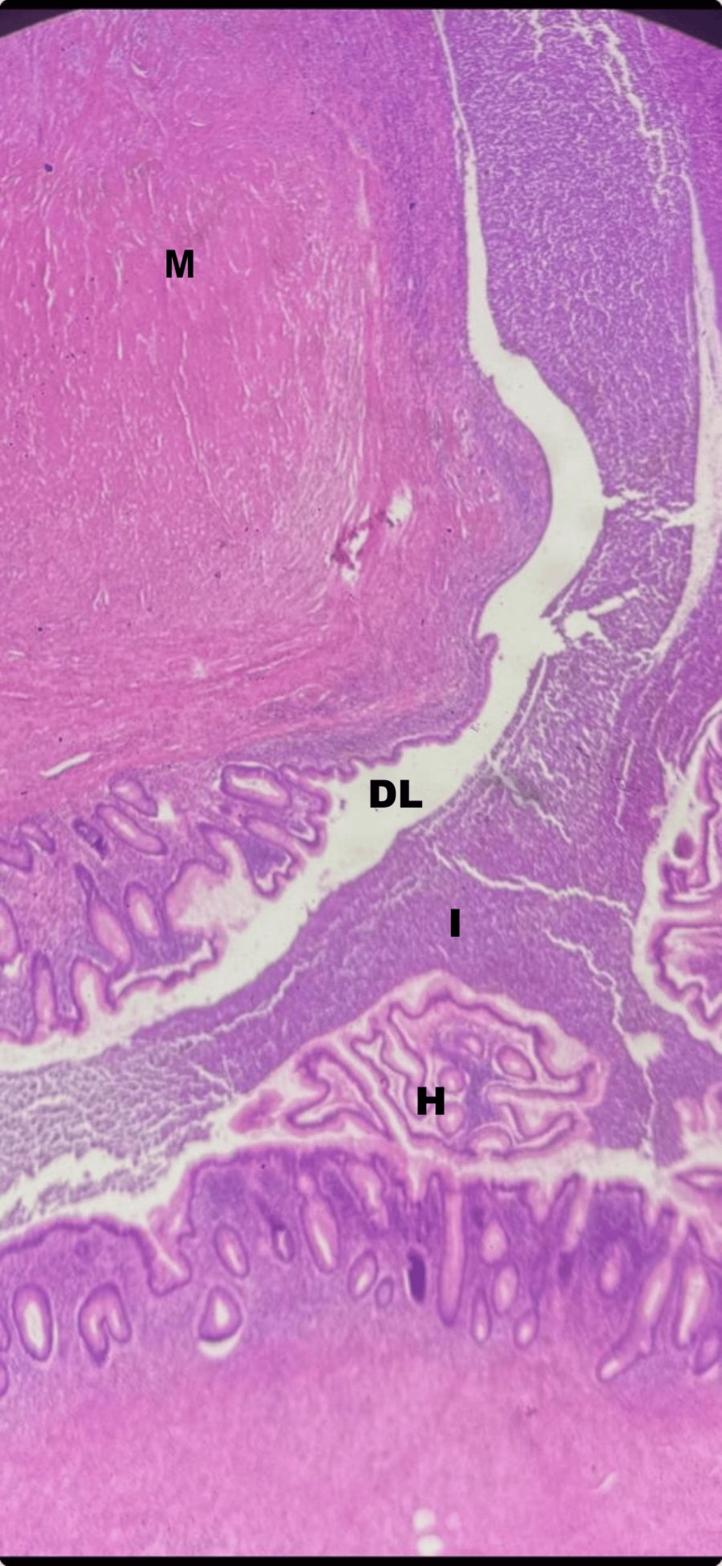
Histopathological section of the appendix (H&E stain, low power) - image 2 The section highlights the key features of a ruptured appendiceal diverticulum: ​M (mucin): a large area of extravasated mucin within the periappendiceal tissue, resulting from the rupture. ​DL (diverticular lumen): the clear space identifying the herniated lumen through the wall. ​I (Infiltration): dense inflammatory infiltrate and fibrosis, correlating with the "phlegmon" observed on preoperative imaging. ​H (herniated mucosa): Appendiceal mucosa protruding through a defect in the muscularis propria

**Figure 6:**
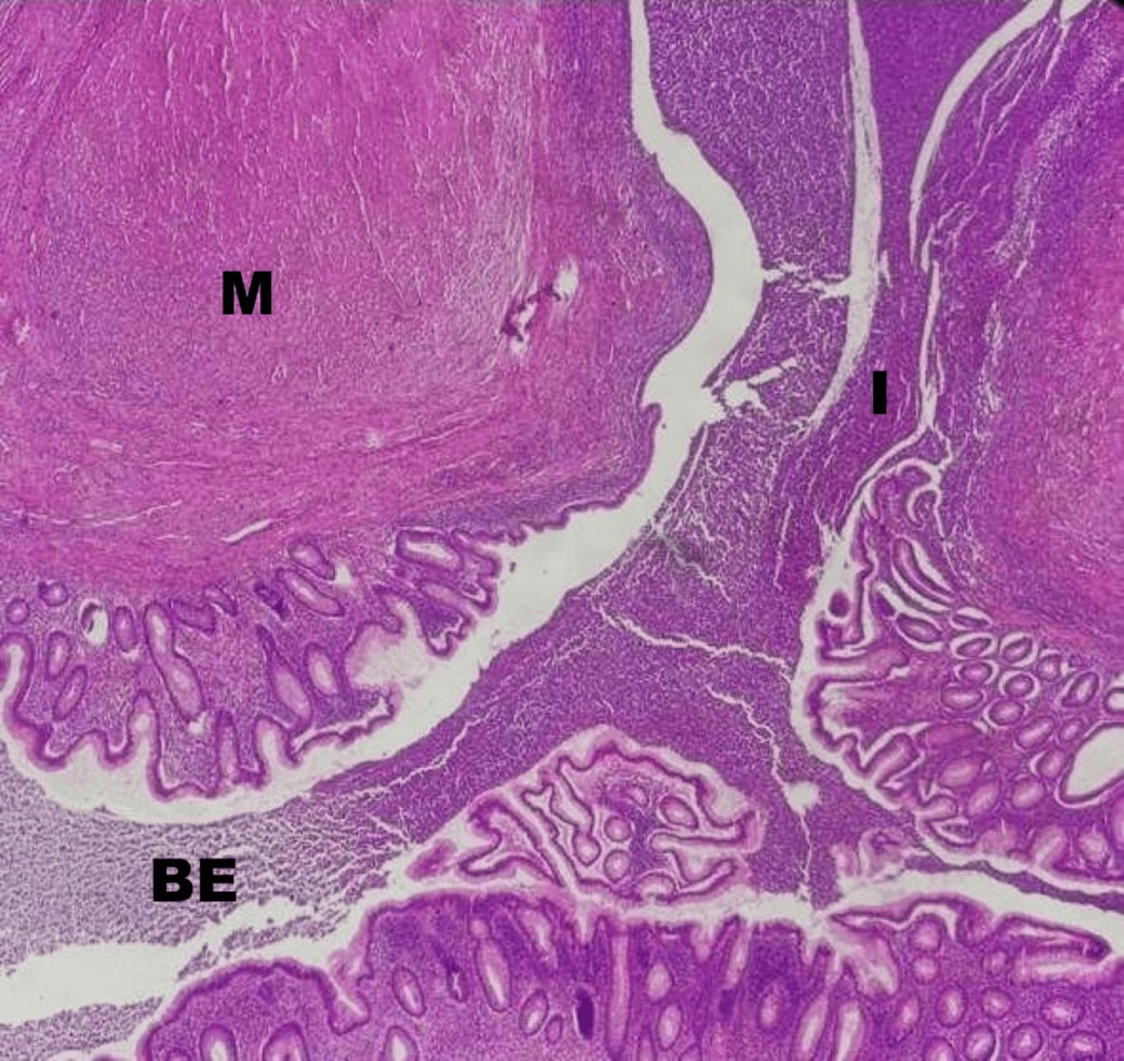
Histopathological examination (H&E stain, high-power) confirming the benign nature of the ruptured diverticulum BE (bland epithelium): points to the mucosal lining of the diverticulum, characterized by uniform, basally located nuclei with preserved polarity and no evidence of nuclear pleomorphism or architectural complexity. ​M (acellular mucin): demonstrates a large area of extravasated, acellular mucin within the periappendiceal soft tissues, resulting from the diverticular rupture. ​I (inflammatory infiltrate): highlights a dense, mixed inflammatory cell infiltrate (predominantly neutrophils and lymphocytes) and reactive fibrosis in response to the perforation

Histopathological examination of the resected appendix revealed a ruptured appendiceal diverticulum with mucin extravasation into the periappendiceal tissue, accompanied by inflammation and fibrosis (Figures 4, 5). No evidence of dysplasia or neoplasia was identified. To further exclude a neoplastic process, high-power microscopic examination of the appendiceal epithelium was performed (Figure 6), confirming a benign, cytologically bland epithelial lining without nuclear pleomorphism, stratification, or complex architecture. These findings effectively ruled out a low-grade appendiceal mucinous neoplasm (LAMN).

## Discussion

Appendiceal diverticulosis is an uncommon condition identified in a small percentage of appendectomy specimens [1,2,5]. It can be congenital, as a true diverticulum involving all layers of the wall, or acquired, as a pseudo-diverticulum with herniation of mucosa and submucosa through the muscularis. Most reported cases are acquired and are frequently discovered incidentally after an appendectomy performed for suspected acute appendicitis [3,6]. Clinically, appendiceal diverticulitis closely resembles acute appendicitis, often presenting with right lower quadrant abdominal pain, fever, and leukocytosis [3,5]. Several studies have reported that symptoms may last longer or be less severe than in typical appendicitis, which can result in delayed presentation [6]. In our case, the patient experienced a three-day history of progressively worsening pain with localized peritoneal signs, matching previously reported clinical patterns.

Radiological diagnosis remains challenging. CT findings often show periappendiceal fat stranding, a localized inflammatory mass, wall thickening, or phlegmon formation [7,8]. Direct identification of a diverticulum on imaging is rare [7]. In this case, CT findings suggested complicated appendicitis with phlegmon and periappendiceal gas, but the diverticulum was not visible preoperatively. This is consistent with prior reports showing that a definitive diagnosis is usually made through histopathological examination rather than imaging [3,7,8].

A key clinical concern noted in the literature is the increased risk of perforation associated with appendiceal diverticulitis [4,5,9]. The relatively thin wall of diverticula makes them more prone to early rupture compared to typical inflamed appendices. Histopathological analysis of our patient revealed a ruptured diverticulum with mucin extravasation, supporting the concept that diverticular inflammation may have a more aggressive course [9]. Another important consideration is the reported association between appendiceal diverticula and appendiceal neoplasms, including mucinous neoplasms and neuroendocrine tumors [3,4,10]. Although the exact mechanism is unclear, this association emphasizes the need for thorough histopathological evaluation of all appendectomy specimens. In the present case, no dysplasia or malignancy was identified, but careful pathological assessment was crucial to rule out underlying neoplastic processes [10].

Surgical management continues to be the standard treatment for symptomatic appendiceal diverticulitis. Early appendectomy is recommended to reduce the risk of progression to perforation or abscess formation [3,5,6]. Both open and laparoscopic approaches are appropriate depending on the clinical scenario and the surgeon's preference. Our patient underwent open appendectomy due to radiological suspicion of complicated appendicitis with phlegmon formation and experienced an uneventful postoperative course. This case highlights several important points: the diagnostic difficulty of appendiceal diverticulitis, its potential for perforation, and the critical role of histopathological examination in excluding associated neoplasia. Increased awareness of this condition may enhance diagnostic suspicion and support timely management in patients presenting with complicated appendicitis [3,5,7,9].

## Conclusions

Ruptured appendiceal diverticulum is an uncommon but clinically significant condition that can present similarly to acute appendicitis, often making preoperative identification difficult. It carries a higher risk of perforation and other complications, emphasizing the importance of careful assessment of patients presenting with right lower quadrant pain. Surgeons should maintain a high index of suspicion, particularly in adults with atypical features or imaging findings indicating phlegmon or localized inflammation. Histopathological examination of the appendectomy specimen is essential not only to confirm the diagnosis but also to exclude underlying neoplastic changes. Early recognition and timely surgical management remain critical to ensure optimal patient outcomes and reduce the risk of complications.
